# Temporal Trends in Mitral Valve Repair Procedures and Outcomes Among Older U.S. Adults

**DOI:** 10.1016/j.jacadv.2026.103283

**Published:** 2026-09-23

**Authors:** Philipp von Stein, David J. Cohen, Martin B. Leon, Alexandra Popma, Jennifer von Stein, Maria C. Alu, Bjorn Redfors, Batla Falah, Juan F. Granada, Jeffrey J. Popma, Harun Kundi

**Affiliations:** a Cardiovascular Research Foundation, New York, New York, USA; b Department of Cardiology, Heart Center, Faculty of Medicine, University of Cologne, Cologne, Germany; c St. Francis Hospital and Heart Center, Roslyn, New York, USA; d Columbia-Presbyterian Medical Center, New York, New York, USA; e Weill-Cornell University Medical Center, New York, New York, USA; f Beth Israel Deaconess Medical Center, Boston, Massachusetts, USA

**Keywords:** mitral regurgitation, mitral valve repair, mitral valve transcatheter edge-to-edge repair, M-TEER, surgical mitral valve repair, utilization trends

## Abstract

**Background:**

Mitral valve (MV) transcatheter edge-to-edge repair (M-TEER) has reshaped the management of mitral regurgitation in high surgical risk patients. As M-TEER adoption expands, understanding concurrent changes in procedural utilization, patient characteristics, and clinical outcomes is important.

**Objectives:**

The objective of the study was to evaluate temporal trends in procedural utilization, comorbidity burden, and mortality among Medicare beneficiaries undergoing MV repair.

**Methods:**

Medicare beneficiaries undergoing MV repair between 2017 and 2021 were analyzed. Procedure volumes, Elixhauser Comorbidity Index, and mortality at 30-days, 30-days postdischarge, 1-year, and 2-years were assessed. Temporal mortality trends were analyzed using adjusted Cox models with calendar year as a continuous variable.

**Results:**

Among 81,215 beneficiaries, 39,097 underwent M-TEER and 42,118 surgical MV repair. M-TEER utilization increased from 15.0 to 27.6 per 100,000 beneficiaries (+84.0%), whereas surgical MV repair decreased from 29.1 to 20.7 per 100,000 (−28.9%), resulting in a 9.5% overall increase in MV repair use. The Elixhauser Comorbidity Index increased overall (+7.8%), remained stable among M-TEER patients (−0.2%), and decreased among surgical MV repair patients (−5.1%). Overall, adjusted mortality decreased at 30-days (adjusted HR [aHR]: 0.92; 95% CI: 0.89-0.94; *P* < 0.001), postdischarge 30-days (aHR: 0.93; 95% CI: 0.89-0.97; *P* < 0.001), 1-year (aHR: 0.98; 95% CI: 0.97-1.00; *P* = 0.013), and 2-years (aHR: 0.98; 95% CI: 0.97-0.99; *P* < 0.001).

**Conclusions:**

Between 2017 and 2021, M-TEER utilization increased substantially whereas surgical MV repair declined, resulting in increased overall MV repair utilization. Over the same period, adjusted short-term and midterm mortality improved despite a higher overall comorbidity index.

Mitral regurgitation (MR) is one of the most common forms of acquired valvular heart disease, affecting approximately 2% to 3% of the general population and nearly 10% of individuals older than 75 years.^1^ Despite its association with increased mortality and frequent heart failure hospitalizations, only about 15% of patients with moderate or severe MR undergo mitral valve (MV) surgery, primarily due to concerns over high surgical risk.^2^^,^^3^ This treatment gap has led to the development and rapid adoption of MV transcatheter edge-to-edge repair (M-TEER), a less invasive MV repair alternative for patients at elevated surgical risk.^4^^,^^5^

Over the past decade, the use of M-TEER has increased substantially in the United States, accompanied by changes in surgical MV repair volumes.^6^ It remains unclear how population-level MV repair utilization, characteristics of treated patients, and clinical outcomes have evolved during this period. We therefore evaluated temporal trends in MV repair utilization, patient characteristics, and short-term and midterm mortality among Medicare beneficiaries undergoing MV repair.

## Methods

### Data source

This study was designed as a retrospective cohort analysis using the Medicare Provider Analysis and Review database, which provides comprehensive hospitalization and claims information for Medicare beneficiaries from 2016 through 2022. Although Medicare Provider Analysis and Review data were available from 2016 through 2022, the analytic cohort was restricted to beneficiaries undergoing MV repair between 2017 and 2021 to ensure a complete 1-year look-back period for comorbidity and frailty assessment and a minimum 1-year follow-up period after the index procedure for all included beneficiaries.

The analysis was based exclusively on deidentified Medicare claims data obtained under a data use agreement with the Centers for Medicare & Medicaid Services and did not constitute human subjects research; therefore, Institutional Review Board approval was not required.

### Study population

Beneficiaries aged 65 years and older were included in the study if they had at least 1 International Statistical Classification of Diseases-10th Revision (ICD-10), Procedure Coding System code for M-TEER (02UG3JZ) or surgical MV repair (02QG0ZZ, 02UG0JZ, 02UG07Z, 02UG08Z, and 02UG0KZ). Beneficiaries were excluded if they did not have continuous Medicare fee-for-service enrollment during the 1-year baseline period, as assessed using the Medicare Master Beneficiary Summary File. To reflect guideline-driven heart-team decision-making and real-world procedural complexity, patients undergoing concomitant coronary artery bypass grafting, transcatheter aortic valve replacement, or other valve procedures were included; however, patients undergoing MV replacement (surgical or transcatheter) were excluded.

Baseline covariates were determined from inpatient claims using secondary diagnosis codes marked as “present on admission” during the index hospitalization, along with primary and secondary diagnosis codes from all hospitalizations occurring within 1 year before the index admission date for each patient. Race was classified as White, Black, or other. The Elixhauser Comorbidity Index was calculated for each patient to assess comorbid conditions.^7^

Frailty was measured using the Hospital Frailty Risk Score (HFRS), a well-established tool developed in the UK that relies on administrative health data.^8^ This score has been widely used in research, particularly in the United States, where studies have linked it to poorer health outcomes, including cardiovascular diseases.^9^ To calculate the HFRS, we examined 109 ICD-10 diagnostic codes from inpatient claims, including hospitalizations that occurred within 1 year before the index admission and secondary diagnoses marked as “present on admission” during the index hospitalization for MV repair (Supplemental Table 1). Based on previously established thresholds, patients were classified into 3 frailty risk categories: low risk (HFRS <5), intermediate risk (HFRS 5-15), or high risk (HFRS >15). Patients with an HFRS ≥5 were considered frail.^8^

For the analysis of procedural utilization trends, we included all MV repair procedures performed between 2017 and 2021, allowing multiple procedures per beneficiary to reflect real-world use patterns.

### Outcomes

Outcome measures included all-cause mortality, which was assessed at 30-days, 1 year, and 2-year follow-up. In addition, we assessed mortality between hospital discharge and 30-days after discharge. Death information was obtained from the Medicare Master Beneficiary Summary File, with follow-up through December 2023. For 30-day, 1-year, and 2-year mortality, follow-up time was calculated from the date of the index procedure. For postdischarge 30-day mortality, the analysis was restricted to patients alive at discharge, and follow-up time was calculated from the date of hospital discharge.

### Statistical analysis

Continuous variables are summarized as mean with SD, while categorical variables are reported as count and percentage. To illustrate trends over time, we plotted the annual use of MV repair based on the volume of each procedure type, which is reported as number per 100,000 Medicare beneficiaries.

We calculated the percentage change in comorbidity prevalence between 2017 and 2021 for the overall MV repair cohort and separately for the M-TEER and surgical MV repair cohorts. We defined a significant change as a ≥10% difference in comorbidity prevalence.^10^ To assess overall trends in comorbidity burden, we also calculated the mean change in the Elixhauser Comorbidity Index within each treatment group. In addition, we performed a complementary annual trend analysis using logistic regression models with each comorbidity as the dependent variable and calendar year (treated as a continuous variable) as the independent predictor. From these models, we calculated the OR per year, which reflects the average yearly increase or decrease in comorbidity prevalence. We also estimated the absolute yearly change in prevalence by calculating the marginal effect of calendar year, which provides the annualized risk difference. Models were run for the overall cohort and separately for M-TEER and surgical MV repair patients. Results are displayed using a dot plot to illustrate the direction of comorbidity changes over the 5-year study period.

To analyze trends in clinical outcomes over time, we used Cox proportional hazards models with the year of intervention as the primary independent variable (ranging from 1 to 5, corresponding to the years 2017 through 2021). These models adjusted for age, sex, race, individual Elixhauser comorbidity indicators, HFRS, and hospital procedural volume. To address potential clustering within institutions, hospitals were included as a random intercept. For models evaluating postdischarge 30-day mortality, we included only patients who were alive at discharge. For each outcome, we report the adjusted HR (aHR) per calendar year to evaluate trends over time. In sensitivity analyses, calendar year was modeled as a categorical variable, with 2017 as the reference, to assess year-specific changes without assuming a log-linear temporal trend. The proportional hazards assumption was assessed using Schoenfeld residuals, with no evidence of violation for calendar year. To assess potential collinearity between the HFRS and individual Elixhauser comorbidity indicators, variance inflation factors were examined. All variance inflation factors were below 1.5, indicating no evidence of meaningful collinearity among model covariates. All statistical analyses were performed in STATA MP (version 17.0, Stata Corporation) or SAS (version 9.4, SAS Institute) using a 2-tailed alpha <0.05 to define statistical significance.

## Results

### Temporal trends in procedural utilization

Between 2017 and 2021, the annual number of MV repair procedures increased from 44.1 to 48.3 per 100,000 beneficiaries, representing a 9.5% overall increase. Among these, M-TEER procedures increased from 15.0 to 27.6 per 100,000 patients, representing an 84.0% increase. In contrast, surgical MV repair decreased from 29.1 to 20.7 per 100,000 patients, corresponding to a 28.9% decline (Figure 1).

**Figure 1:**
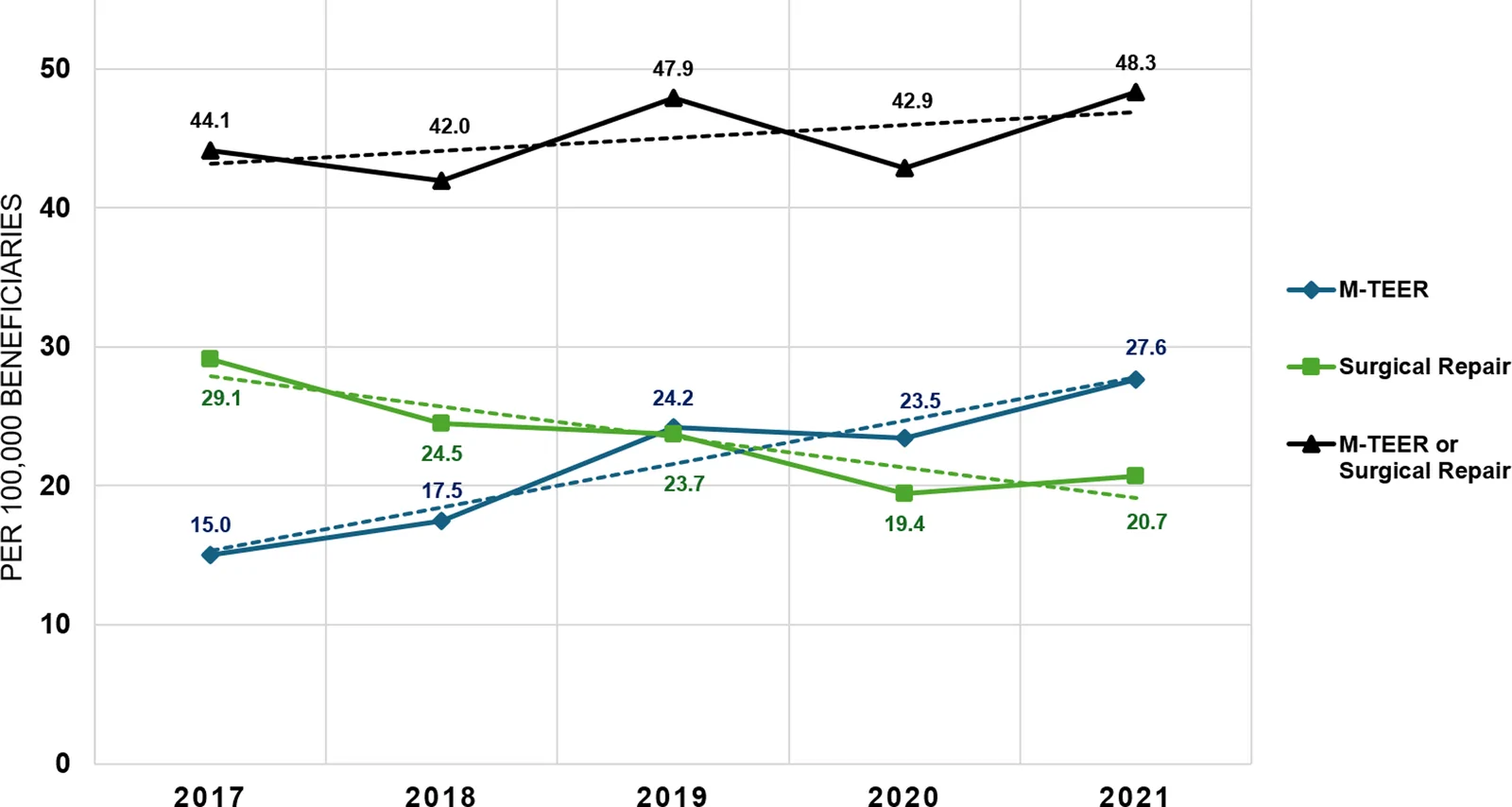
Annual Utilization Trends by Mitral Valve Repair Type The dashed lines represent linearized trends. M-TEER = mitral valve transcatheter edge-to-edge repair.

### Temporal trends in patient characteristics

Over the study period, the mean age of all patients undergoing MV repair increased slightly (from 76.1 to 76.8 years) (Table 1). In contrast, within each treatment group, the mean age decreased modestly—from 80.6 to 79.6 years for M-TEER and from 73.7 to 73.0 years for surgical MV repair (Supplemental Tables 2 and 3).

**Table 1:** Temporal Trends in Baseline Characteristics of Patients Undergoing Mitral Valve Repair

	2017 (n = 15,028)	2018 (n = 14,738)	2019 (n = 17,504)	2020 (n = 15,935)	2021 (n = 18,010)
Age, y	76.1 ± 7.2	76.4 ± 7.3	76.7 ± 7.3	76.5 ± 7.3	76.8 ± 7.2
Male	8,540 (56.8)	8,470 (57.5)	10,055 (57.4)	9,117 (57.2)	10,091 (56.0)
Race					
White	13,289 (88.4)	12,983 (88.1)	15,232 (87.0)	13,902 (87.2)	15,603 (86.6)
Black	816 (5.4)	799 (5.4)	1,058 (6.0)	989 (6.2)	1,131 (6.3)
Other	924 (6.1)	956 (6.5)	1,214 (6.9)	1,046 (6.6)	1,276 (7.1)
Congestive heart failure	10,435 (69.4)	10,584 (71.8)	13,024 (74.4)	12,115 (76.0)	13,619 (75.6)
Cardiac arrhythmias	9,457 (62.9)	9,563 (64.9)	11,464 (65.5)	10,605 (66.5)	11,939 (66.3)
Atrial fibrillation	7,864 (52.3)	7,999 (54.3)	9,535 (54.5)	8,781 (55.1)	9,892 (54.9)
Pulmonary circulation disorders	5,173 (34.4)	5,117 (34.7)	6,020 (34.4)	5,423 (34.0)	5,885 (32.7)
Peripheral vascular disorders	2,798 (18.6)	2,810 (19.1)	3,474 (19.8)	3,339 (21.0)	3,536 (19.6)
Hypertension	9,208 (61.3)	9,730 (66.0)	12,031 (68.7)	11,230 (70.5)	12,582 (69.9)
Paralysis	152 (1.0)	128 (0.9)	146 (0.8)	147 (0.9)	151 (0.8)
Other neurological disorders	893 (5.9)	873 (5.9)	1,100 (6.3)	1,101 (6.9)	1,233 (6.8)
Chronic pulmonary disease	4,066 (27.1)	4,002 (27.2)	4,756 (27.2)	4,317 (27.1)	4,630 (25.7)
Diabetes, uncomplicated	2,092 (13.9)	1,801 (12.2)	2,111 (12.1)	1,843 (11.6)	1,944 (10.8)
Diabetes, complicated	2,611 (17.4)	2,688 (18.2)	3,353 (19.2)	3,105 (19.5)	3,358 (18.6)
Hypothyroidism	2,924 (19.5)	2,840 (19.3)	3,632 (20.7)	3,229 (20.3)	3,647 (20.2)
Renal failure	4,811 (32.0)	4,958 (33.6)	6,175 (35.3)	5,837 (36.6)	6,548 (36.4)
Liver disease	523 (3.5)	528 (3.6)	749 (4.3)	765 (4.8)	899 (5.0)
Peptic ulcer disease excluding bleeding	199 (1.3)	150 (1.0)	203 (1.2)	172 (1.1)	178 (1.0)
AIDS/HIV	14 (0.1)	17 (0.1)	13 (0.1)	13 (0.1)	13 (0.1)
Lymphoma	142 (0.9)	117 (0.8)	212 (1.2)	153 (1.0)	205 (1.1)
Metastatic cancer	86 (0.6)	90 (0.6)	138 (0.8)	115 (0.7)	131 (0.7)
Solid tumor without metastasis	402 (2.7)	411 (2.8)	616 (3.5)	528 (3.3)	593 (3.3)
Rheumatoid arthritis/collagen vascular disorder	620 (4.1)	669 (4.5)	795 (4.5)	687 (4.3)	766 (4.3)
Coagulopathy	2,090 (13.9)	2,011 (13.6)	2,406 (13.7)	2,273 (14.3)	2,564 (14.2)
Obesity	2,510 (16.7)	2,514 (17.1)	3,046 (17.4)	2,936 (18.4)	3,355 (18.6)
Weight loss	1,051 (7.0)	1,049 (7.1)	1,338 (7.6)	1,240 (7.8)	1,300 (7.2)
Fluid and electrolyte disorders	3,738 (24.9)	3,843 (26.1)	4,748 (27.1)	4,700 (29.5)	4,974 (27.6)
Blood loss anemia	263 (1.7)	274 (1.9)	343 (2.0)	265 (1.7)	269 (1.5)
Deficiency anemia	963 (6.4)	1,058 (7.2)	1,349 (7.7)	1,316 (8.3)	1,479 (8.2)
Alcohol abuse	353 (2.3)	359 (2.4)	405 (2.3)	408 (2.6)	460 (2.6)
Drug abuse	123 (0.8)	179 (1.2)	215 (1.2)	228 (1.4)	192 (1.1)
Psychoses	47 (0.3)	33 (0.2)	42 (0.2)	50 (0.3)	56 (0.3)
Depression	1,781 (11.9)	1,719 (11.7)	2,180 (12.5)	2,033 (12.8)	2,340 (13.0)
Hospital Frailty Risk Score	4.4 ± 5.7	4.7 ± 5.9	4.9 ± 6.0	5.0 ± 6.1	4.7 ± 5.8
Frailty risk					
Low	10,347 (68.8)	9,883 (67.1)	11,525 (65.8)	10,204 (64.0)	11,961 (66.4)
Intermediate	3,784 (25.2)	3,861 (26.2)	4,692 (26.8)	4,545 (28.5)	4,852 (26.9)
High	898 (6.0)	994 (6.7)	1,287 (7.4)	1,188 (7.5)	1,197 (6.6)
Frail	4,682 (31.2)	4,855 (32.9)	5,979 (34.2)	5,733 (36.0)	6,049 (33.6)

Values are mean ± SD or n (%).

Figure 2 demonstrates the change in comorbidity prevalence by procedure type over the study period. In the overall cohort, the Elixhauser Comorbidity Index increased modestly with a mean relative change of +7.8%. When evaluated by treatment group, comorbidity burden remained essentially stable among patients undergoing M-TEER (mean change −0.2%), whereas those treated with surgical MV repair experienced a more pronounced reduction (−5.1%). Among clinically relevant comorbidities, the prevalence of congestive heart failure increased from 69.4% to 75.6%, renal failure from 32.0% to 36.4%, hypertension from 61.3% to 69.9%, and liver disease from 3.5% to 5.0%. In sensitivity analyses, logistic regression models evaluating annual changes in comorbidities and the corresponding annualized risk-difference estimates demonstrated temporal trends that were consistent with the main analysis across all treatment groups (Supplemental Figures 1 and 2).

**Figure 2:**
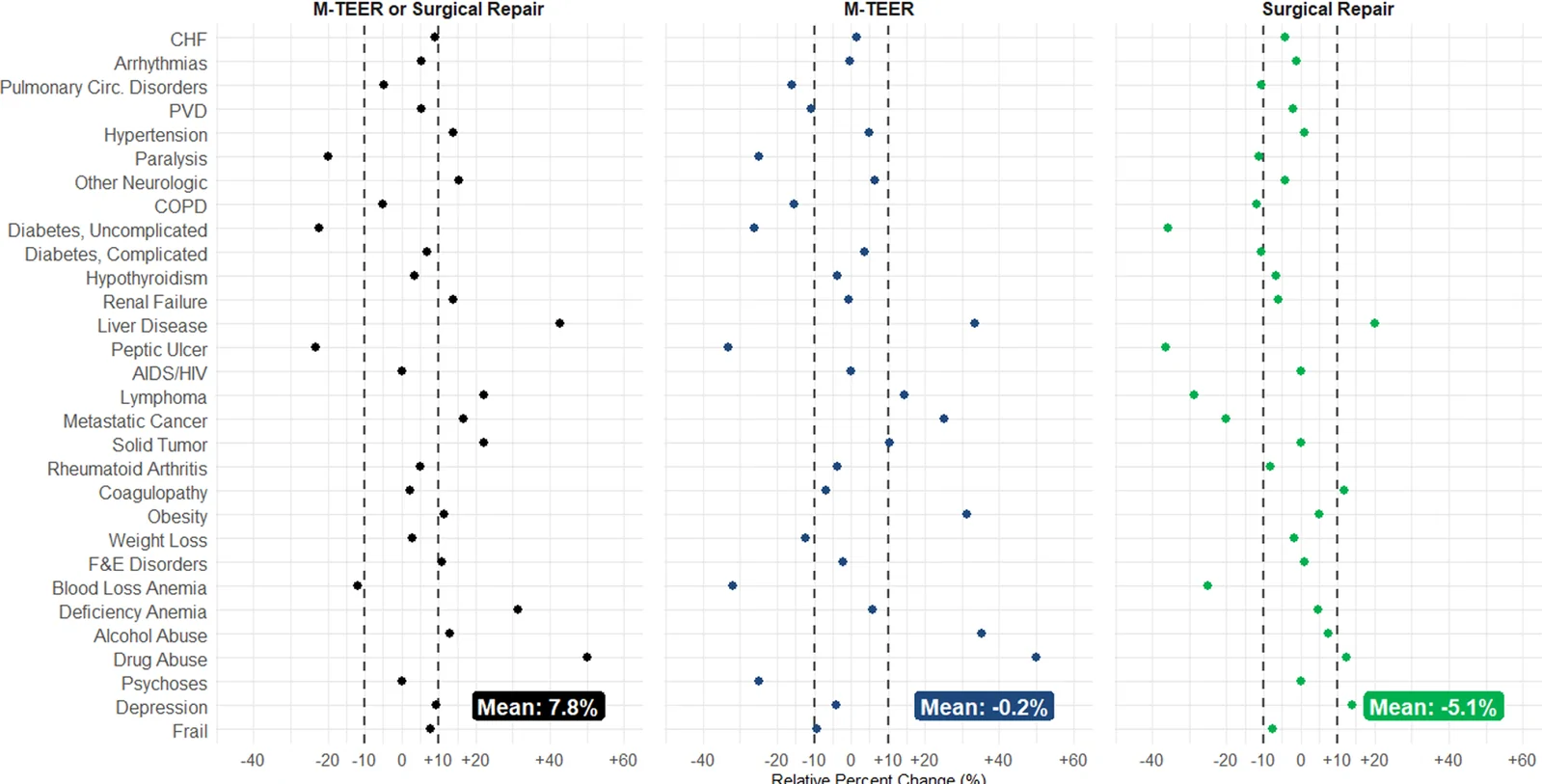
Relative Percent Change in Comorbidity Prevalence by Mitral Valve Repair Type Between 2017 and 2021 Dashed vertical lines at the +10% and −10% thresholds indicate the prespecified levels considered statistically significant. CHF = congestive heart failure; COPD = chronic obstructive pulmonary disease; F&E = fluid and electrolyte; PVD = peripheral vascular disease; other abbreviation as in Figure 1.

### Temporal trends in unadjusted outcomes

In the overall cohort, gradual and sustained reductions in mortality were observed across all follow-up time points during the study period (Table 2). The 30-day mortality rate declined from 4.1% to 3.0% in the M-TEER group and from 4.4% to 3.7% in the surgical MV repair group, with similar trends for postdischarge 30-day mortality, which decreased from 2.8% to 2.1% in the M-TEER group and from 1.8% to 1.2% in the surgical MV repair group. Improvements were also observed in midterm survival. The 1-year mortality rate declined from 21.2% to 18.8% in M-TEER and from 9.8% to 8.1% in surgical MV repair. 2-year mortality followed a similar trend, decreasing from 33.6% to 28.7% in patients undergoing M-TEER and from 13.2% to 10.0% in patients undergoing surgical MV repair.

**Table 2:** Unadjusted Outcomes Among Patients Undergoing Mitral Valve Repair

M-TEER or Surgical MV Repair	2017 (n = 15,028)	2018 (n = 14,738)	2019 (n = 17,504)	2020 (n = 15,935)	2021 (n = 18,010)
30-day mortality	4.3 [4.0-4.6]	3.8 [3.5-4.2]	3.7 [3.4-4.0]	3.6 [3.3-3.9]	3.3 [3.1-3.6]
Postdischarge 30-day mortality^a^	2.1 [1.9-2.3]	2.2 [2.0-2.4]	1.9 [1.7-2.2]	1.9 [1.7-2.2]	1.7 [1.5-1.9]
1-year mortality	13.7 [13.1-14.2]	13.6 [13.1-14.2]	14.7 [14.2-15.2]	15.3 [14.8-15.9]	14.2 [13.7-14.8]
2-year mortality	20.1 [19.5-20.8]	20.6 [20.0-21.3]	22.6 [22.0-23.2]	22.9 [22.2-23.6]	20.7 [20.1-21.7]

**M-TEER**	**(n = 5,118)**	**(n = 6,138)**	**(n = 8,833)**	**(n = 8,707)**	**(n = 10,301)**

30-day mortality	4.1 [3.6-4.7]	4.1 [3.7-4.7]	3.8 [3.4-4.2]	3.2 [2.8-3.6]	3.0 [2.7-3.4]
Postdischarge 30-day mortality^a^	2.8 [2.3-3.2]	3.0 [2.5-3.4]	2.5 [2.2-2.9]	2.2 [1.9-2.5]	2.1 [1.8-2.4]
1-year mortality	21.2 [20.1-22.3]	20.5 [19.5-21.6]	20.9 [20.1-21.8]	20.1 [19.3-21.0]	18.8 [18.1-19.6]
2-year mortality	33.6 [32.3-34.9]	33.0 [31.8-34.2]	33.8 [32.8-34.8]	31.9 [30.9-32.9]	28.7 [27.8-29.6]

**Surgical MV Repair**	**(n = 9,910)**	**(n = 8,600)**	**(n = 8,671)**	**(n = 7,228)**	**(n = 7,709)**

30-day mortality	4.4 [4.0-4.8]	3.6 [3.2-4.0]	3.5 [3.2-4.0]	4.0 [3.6-4.5]	3.7 [3.3-4.1]
Postdischarge 30-day mortality^a^	1.8 [1.5-2.0]	1.6 [1.4-1.9]	1.3 [1.1-1.6]	1.6 [1.3-1.9]	1.2 [1.0-1.5]
1-year mortality	9.8 [9.2-10.4]	8.7 [8.1-9.3]	8.3 [7.7-8.9]	9.5 [8.8-10.2]	8.1 [7.5-8.7]
2-year mortality	13.2 [12.5-13.8]	11.8 [11.1-12.5]	11.2 [10.5-11.9]	12.1 [11.4-12.9]	10.0 [9.3-10.7]

Values are event rate [95% CI].

M-TEER = mitral valve transcatheter edge-to-edge repair; MV = mitral valve.

a Rates based on patients alive at discharge.

### Temporal trends in adjusted outcomes

In the overall cohort, significant improvements were observed for risk-adjusted mortality at all follow-up time points including 30-day follow-up (aHR per calendar year: 0.92, 95% CI: 0.89-0.94; *P* < 0.001), postdischarge 30-day follow-up (aHR: 0.93; 95% CI: 0.89-0.97; *P* < 0.001), 1-year follow-up (aHR: 0.98; 95% CI: 0.97-1.00; *P* = 0.013), and 2-year follow-up (aHR: 0.98; 95% CI: 0.97-0.99; *P* < 0.001) (Figure 3). When analyzed by treatment group, patients undergoing M-TEER demonstrated reductions in mortality for 30-day follow-up (aHR: 0.91; 95% CI: 0.88-0.95; *P* < 0.001), postdischarge 30-day follow-up (aHR: 0.94; 95% CI: 0.89-0.99; *P* = 0.044), 1-year follow-up (aHR: 0.97, 95% CI: 0.95-0.99; *P* < 0.001), and 2-year follow-up (aHR: 0.95; 95% CI: 0.94-0.97; *P* < 0.001). Surgical MV repair showed no significant improvement in risk-adjusted 30-day (aHR: 0.98; 95% CI: 0.94-1.01; *P* = 0.244) or 1-year mortality (aHR: 0.98, 95% CI: 0.96-1.01; *P* = 0.133) over the study period. However, there was a significant reduction in risk-adjusted postdischarge 30-day mortality (aHR: 0.93; 95% CI: 0.87-0.99; *P* = 0.024) and 2-year mortality (aHR: 0.96; 95% CI: 0.94-0.98; *P* < 0.001). In sensitivity analyses treating calendar year categorically, year-specific estimates generally demonstrated lower mortality in later calendar years, although the magnitude of improvement was not uniform across years. For example, in the overall cohort, the aHRs for 2021 vs 2017 were 0.78 (95% CI: 0.70-0.88) for 30-day mortality, 0.87 (95% CI: 0.81-0.92) for 1-year mortality, and 0.82 (95% CI: 0.77-0.86) for 2-year mortality.

**Figure 3:**
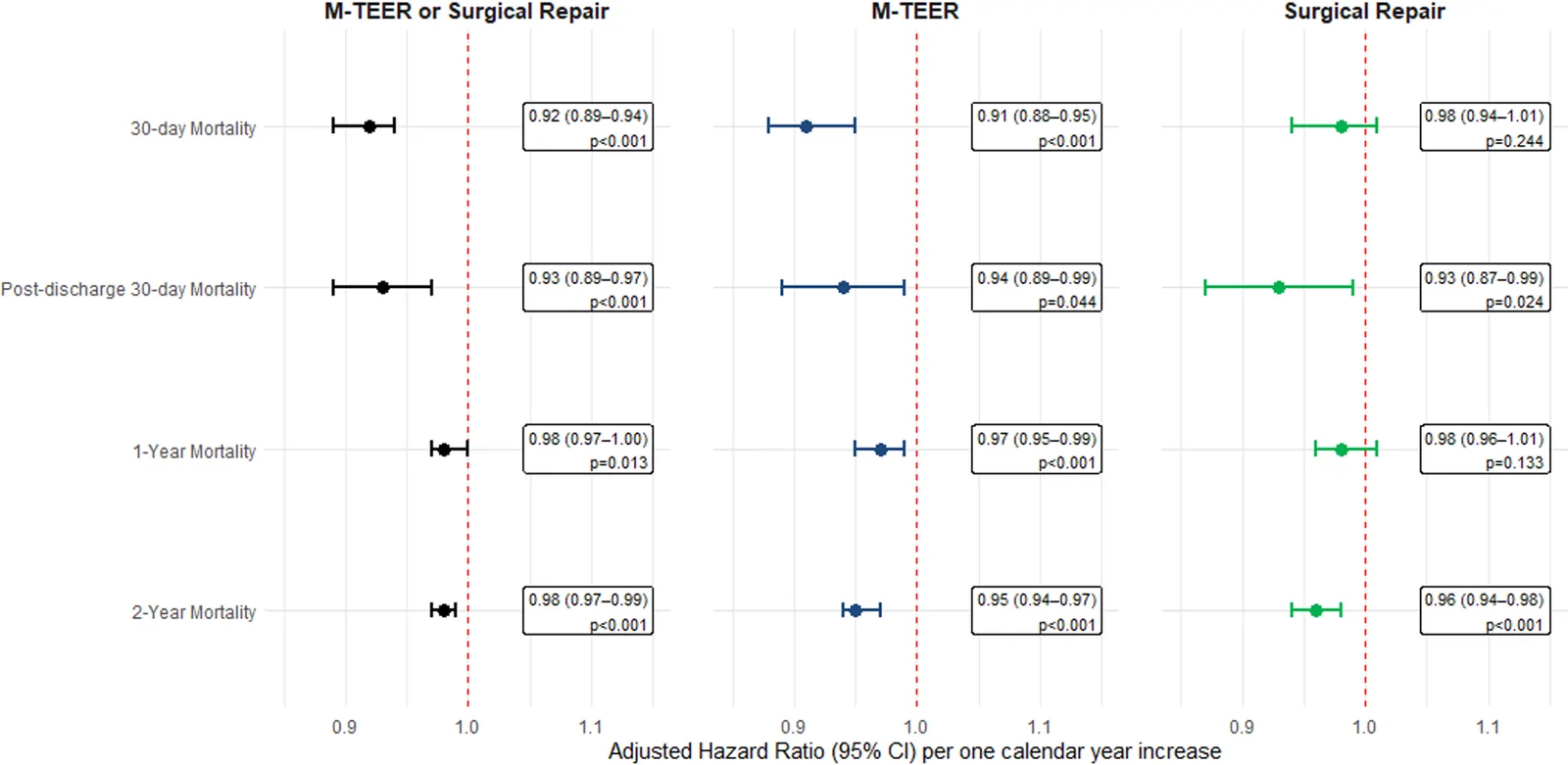
Annual Changes in the Adjusted Risk of Outcomes by Mitral Valve Repair Type Abbreviation as in Figure 1.

## Discussion

In this large, nationally-representative cohort of Medicare beneficiaries undergoing MV repair between 2017 and 2021, we observed substantial temporal changes in procedural utilization, patient characteristics, and clinical outcomes. First, overall utilization of MV repair in the Medicare population increased, driven by a substantial increase in M-TEER (84.0%) use and a concurrent decline (28.9%) in surgical MV repair. Second, in the overall Medicare population, patients undergoing MV repair during the study period became older and exhibited a higher overall comorbidity burden. Third, despite a higher overall comorbidity index over time, risk-adjusted mortality for the overall cohort improved, with significant reductions in 30-day, postdischarge 30-day, 1-year, and 2-year mortality. Significant temporal reductions in mortality were also observed among patients undergoing M-TEER.

Our findings provide insights into temporal changes in procedural utilization, characteristics of the treated population, and clinical outcomes during a period of rapid expansion of M-TEER. Over the 5-year study period, the composition of the treated population changed substantially, reflecting the increasing proportion of patients undergoing M-TEER and a concurrent decline in surgical MV repair. Accordingly, the slight increase in the mean age in the overall cohort, despite modest decreases within both treatment groups, appears to reflect the compositional shift toward M-TEER, whose recipients were consistently older than patients undergoing surgical MV repair. Similarly, the higher overall Elixhauser Comorbidity Index over time, despite an essentially stable index among M-TEER recipients and a decrease among surgical MV repair patients, appears to reflect in part the increasing proportion of M-TEER recipients, who had a higher comorbidity burden than patients undergoing surgical MV repair throughout the study period. These shifts in treatment patterns occurred concurrently with reductions in unadjusted short- and long-term mortality among both patients undergoing surgical MV repair and those undergoing M-TEER. Importantly, the increase in overall MV repair utilization occurred concurrently with improvements in short- and intermediate-term mortality, providing reassurance that the changing procedural landscape was not accompanied by worsening population-level mortality among treated Medicare beneficiaries. (Central Illustration).

**Central Illustration:**
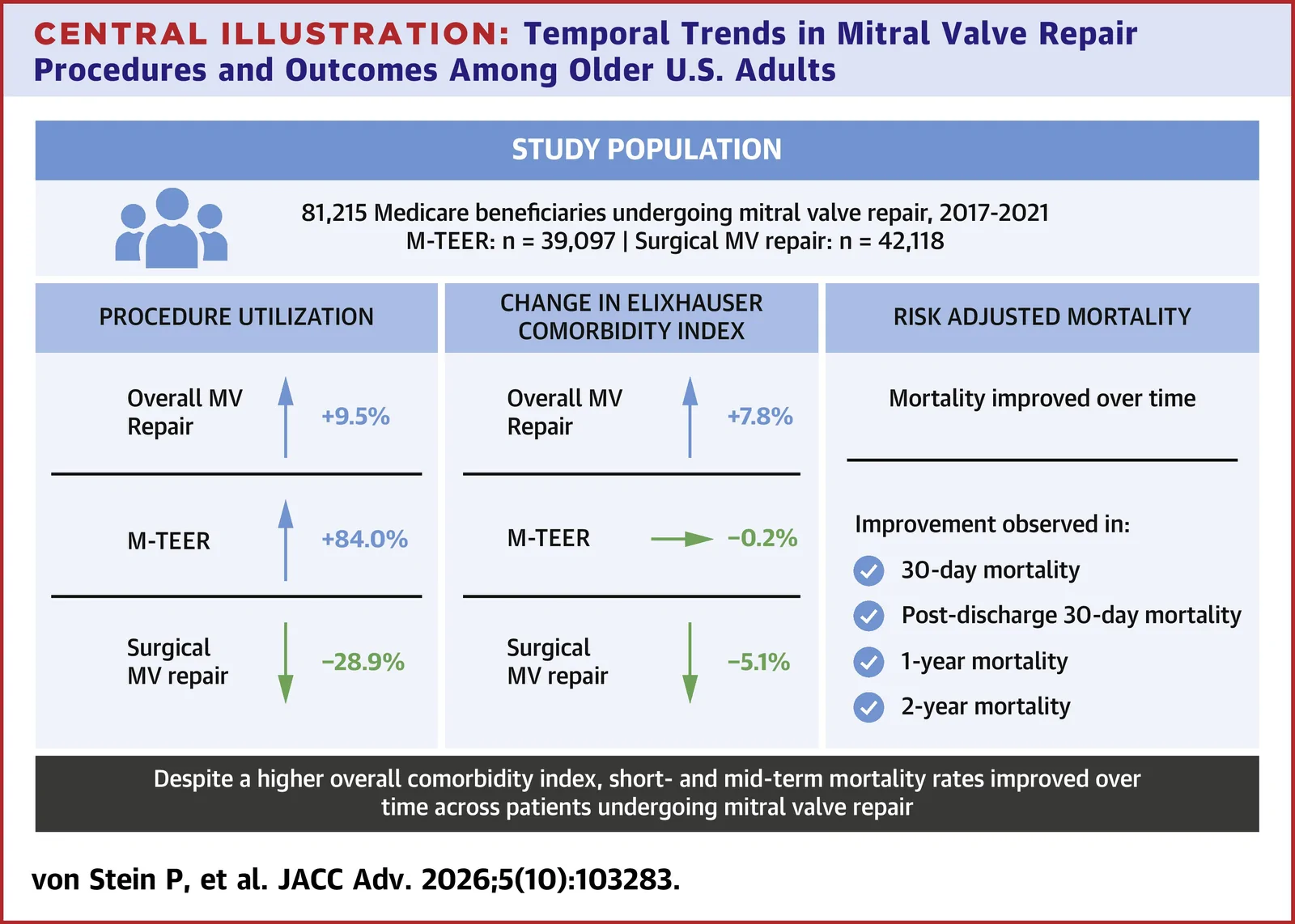
Temporal Trends in Mitral Valve Repair Procedures and Outcomes Among Older U.S. Adults Among Medicare beneficiaries undergoing MV repair between 2017 and 2021, M-TEER utilization increased whereas surgical MV repair declined, resulting in an overall increase in MV repair use. The mean Elixhauser Comorbidity Index was higher in the overall treated cohort over time, while adjusted short- and mid-term mortality improved. M-TEER = mitral valve transcatheter edge-to-edge repair; MV= mitral valve.

Several additional factors may plausibly have contributed to the observed temporal improvement in outcomes among patients undergoing M-TEER.^11^, ^12^, ^13^, ^14^ These include increasing operator and institutional experience, procedural standardization, iterative device refinement, and advances in imaging guidance, and periprocedural management. Although these factors cannot be directly measured in administrative data, the consistent improvement in short- and intermediate-term outcomes observed is directionally consistent with the maturation of M-TEER within routine clinical practice.

In addition, regulatory approval of M-TEER for secondary MR by the U.S. Food and Drug Administration in 2019—based on the COAPT (Cardiovascular Outcomes Assessment of the MitraClip Percutaneous Therapy for Heart Failure Patients With Functional Mitral Regurgitation) trial—likely influenced clinical practice.^15^ This expanded indication may have contributed to the observed increase in M-TEER utilization, particularly among patients with secondary MR who are frequently considered high-risk for surgery.^16^

Our findings should also be interpreted in the context of contemporary Society of Thoracic Surgeons-American College of Cardiology Transcatheter Valve Therapy Registry Registry data. In a recent analysis of 68,028 patients undergoing M-TEER, overall 30-day mortality was 2.5%, whereas 1-year mortality ranged from 17.1% to 21.5% across MR mechanisms.^17^ In comparison, 30-day mortality in our M-TEER cohort declined from 4.1% to 3.0% and 1-year mortality from 21.2% to 18.8% between 2017 and 2021. The somewhat higher early mortality observed in our cohort may reflect differences in case selection, as the transcatheter valve therapy analysis excluded patients with shock, preprocedural inotrope use, or mechanical circulatory support, whereas our Medicare cohort was designed to capture a broader population of older patients undergoing M-TEER. Despite these differences, the broadly similar 1-year mortality rates across the 2 data sources provide reassurance regarding the generalizability of our findings.

The temporal improvement in outcomes among patients undergoing surgical MV repair occurred alongside a reduction in comorbidity burden within the surgical cohort. This pattern is consistent with, but does not establish, changing treatment selection as M-TEER utilization increased. Our findings are consistent with prior evidence demonstrating that the introduction of M-TEER at surgical centers was associated with a decrease in the number of high-risk patients undergoing surgical MV repair at those institutions with an associated improvement in short-term surgical outcomes.^18^ In contrast, the mean Elixhauser Comorbidity Index remained essentially stable within the M-TEER group (−0.2%) over the study period. These trends align with the broader shift toward individualized MV care and reinforce the value of multidisciplinary heart team assessment. As adoption of M-TEER has expanded, outcomes among treated patients improved over the study period, consistent with the continued maturation of M-TEER in contemporary practice.^4^^,^^5^^,^^19^

### Study limitations

Our study has several important limitations. First, the analysis was restricted to Medicare fee-for-service beneficiaries and did not include Medicare Advantage enrollees, which may limit generalizability to the broader population of older Americans. Because Medicare Advantage enrollment changed substantially during the study period, shifts between fee-for-service and Medicare Advantage may also have influenced population-level utilization and outcome trends. Because administrative claims do not capture MR severity, valve anatomy, symptoms, or procedural candidacy, we could not reliably identify the population clinically eligible for MV repair; accordingly, population-level procedure rates should not be interpreted as estimates of treatment uptake among eligible patients. The study period should also be interpreted in the context of the evolving MV treatment landscape, as it does not capture the early adoption phase following the 2013 U.S. Food and Drug Administration approval of M-TEER for primary MR or more recent changes after 2021 in device technology, procedural experience, and patient selection. Second, comorbidities and frailty were identified from inpatient claims using ICD-10 codes rather than standardized prospective definitions. Because carrier/professional claims were not available, outpatient-managed conditions may have been underascertained, potentially underestimating comorbidity and frailty burden. Although ascertainment was consistent across years and treatment groups, changes in coding practices, hospitalization patterns, or patient selection may have influenced observed comorbidity trends. Third, the level of granularity in claims data did not allow us to calculate traditional surgical risk scores, such as EuroSCORE or STS-PROM, or to reliably classify MR etiology. Therefore, temporal trends could not be stratified by primary, secondary, or mixed MR mechanisms. Fourth, patients undergoing concomitant cardiac procedures, including coronary artery bypass grafting or other valve interventions, were included to reflect real-world, guideline-driven clinical decision-making within multidisciplinary heart teams. Although this approach enhances external validity, it may introduce additional heterogeneity in procedural complexity and periprocedural risk that cannot be fully captured through claims-based adjustment. Fifth, the study period overlapped with the COVID-19 pandemic, which may have influenced hospitalization patterns, referral practices, coding behavior, and short-term outcomes in ways not fully captured by claims data. Although calendar year was modeled to account for secular trends, residual confounding related to pandemic-associated disruptions cannot be excluded. Sixth, we did not have access to medication information which may have contributed to improved outcomes over time. Seventh, we did not have access to data on health status and symptomatic outcomes, which are often the highest priority for older patients.

Finally, although reassuring, the fact that short- and intermediate-term mortality improved across the full study cohort over the 5-year time frame encompassed by our study does not mean that treatment selection was optimized for all patients. Indeed, it is possible that different approaches to case selection could have resulted in even better outcomes for the full cohort. In addition, long-term follow-up is necessary to determine whether the observed improvements in early- and intermediate-term mortality are durable over a 5- to 10-year time horizon.

## Conclusions

In this population-based study of older Americans undergoing MV repair, M-TEER utilization increased substantially whereas surgical MV repair declined, resulting in increased overall MV repair utilization and a changing composition of the treated population. Over the same period, short- and intermediate-term mortality improved despite a higher overall comorbidity index among treated beneficiaries. Continued population-level assessment of these outcomes will be critical to ensure that further expansion of transcatheter MV repair (and replacement) techniques does not compromise long-term clinical outcomes for patients with MV disease.

Perspectives **COMPETENCY IN MEDICAL KNOWLEDGE:** In this nationwide Medicare analysis, M-TEER adoption increased substantially between 2017 and 2021, whereas surgical MV repair declined, resulting in an overall increase in MV repair utilization. Despite a higher overall Elixhauser Comorbidity Index over time, short-term and midterm mortality improved among treated beneficiaries.

**COMPETENCY IN SYSTEMS-BASED PRACTICE:** Population-level monitoring of treatment patterns and outcomes is essential as transcatheter MV therapies expand, particularly as procedural volumes and the characteristics of treated patients continue to evolve.

**TRANSLATIONAL OUTLOOK:** Further studies are needed to determine whether these favorable temporal trends persist in more contemporary practice, particularly with continued device iteration, increasing procedural experience, evolving patient selection, and emerging transcatheter MV replacement technologies.

## Funding support and author disclosures

Dr Cohen has received institutional research support from Abbott Laboratories, Edwards Lifesciences, Boston Scientific, Philips, and Zoll Medical; and has received consulting income from Abbott Laboratories, Edwards Lifesciences, Boston Scientific, Medtronic, and Elixir Medical. Dr Leon has received institutional research support from Edwards Lifesciences, Medtronic, Boston Scientific, and Abbott Laboratories; and is a consultant or advisory board member for Foldax, Anteris, JenaValve, Medinol, SoloPace, and Bain Capital. Dr Redfors has received consulting fees from Pfizer and Boehringer Ingelheim. Dr Granada is the cofounder of Cephea Valve Technologies (Abbott Laboratories) and chief executive officer of the Cardio-vascular Research Foundation. Dr J Popma is a former employee of and has nonvested equity in Medtronic. All other authors have reported that they have no relationships relevant to the contents of this paper to disclose.
