# Supplementary material for: Temporal Trends in Mitral Valve Repair Procedures and Outcomes Among Older U.S. Adults
Source: JACC Adv. 2026 Sep 23;5(10):103283. doi: 10.1016/j.jacadv.2026.103283 (PMC13634761; doi:10.1016/j.jacadv.2026.103283)
Supplement: Supplementary file 1 [file mmc1.docx]

# SUPPLEMENTAL MATERIAL

## Table S1. List of ICD-10 Codes, Prevalence and Number of Points for Each to Create the “Hospital Frailty Risk” Score

|  |  | **M-TEER**  **N=39,098** | **Surgical Repair N=42,118** | **Point** |
| --- | --- | --- | --- | --- |
| G81 | Hemiplegia | 272 (0.7) | 274 (0.7) | 4·4 |
| G30 | Alzheimer's disease | 321 (0.8) | 87 (0.2) | 4·0 |
| I69 | Sequelae of cerebrovascular disease (secondary codes) | 738 (1.9) | 339 (0.8) | 3·7 |
| R29 | Other symptoms and signs involving the nervous and musculoskeletal systems (R29·6 Tendency to fall) | 1,057 (2.7) | 604 (1.4) | 3·6 |
| N39 | Other disorders of urinary system (includes urinary tract infection and urinary incontinence) | 3,707 (9.5) | 2,034 (4.8) | 3·2 |
| F05 | Delirium, not induced by alcohol and other psychoactive substances | 433 (1.1) | 152 (0.4) | 3·2 |
| W19 | Unspecified fall | 61 (0.2) | 15 (0.0) | 3·2 |
| S00 | Superficial injury of head | 247 (0.6) | 80 (0.2) | 3·2 |
| R31 | Unspecified hematuria | 927 (2.4) | 518 (1.2) | 3·0 |
| B96 | Other bacterial agents as the cause of diseases classified to other chapters (secondary code) | 1,685 (4.3) | 968 (2.3) | 2·9 |
| R41 | Other symptoms and signs involving cognitive functions and awareness | 1,059 (2.7) | 465 (1.1) | 2·7 |
| R26 | Abnormalities of gait and mobility | 2,370 (6.1) | 775 (1.8) | 2·6 |
| I67 | Other cerebrovascular diseases | 307 (0.8) | 238 (0.6) | 2·6 |
| R56 | Convulsions, not elsewhere classified | 209 (0.5) | 145 (0.3) | 2·6 |
| R40 | Somnolence, stupor and coma | 365 (0.9) | 267 (0.6) | 2·5 |
| T83 | Complications of genitourinary prosthetic devices, implants and grafts | 229 (0.6) | 89 (0.2) | 2·4 |
| S06 | Intracranial injury | 197 (0.5) | 102 (0.2) | 2·4 |
| S42 | Fracture of shoulder and upper arm | 111 (0.3) | 44 (0.1) | 2·3 |
| E87 | Other disorders of fluid, electrolyte and acid-base balance | 12,507 (32.0) | 7,992 (19.0) | 2·3 |
| M25 | Other joint disorders, not elsewhere classified | 837 (2.1) | 532 (1.3) | 2·3 |
| E86 | Volume depletion | 3,054 (7.8) | 1,583 (3.8) | 2·3 |
| R54 | Senility | 1,451 (3.7) | 169 (0.4) | 2·2 |
| F03 | Unspecified dementia | 1,642 (4.2) | 483 (1.1) | 2·1 |
| W18 | Other fall on same level | 24 (0.1) | 7 (0.0) | 2·1 |
| Z75 | Problems related to medical facilities and other health care | 26 (0.1) | 10 (0.0) | 2·0 |
| F01 | Vascular dementia | 138 (0.4) | 41 (0.1) | 2·0 |
| S80 | Superficial injury of lower leg | 178 (0.5) | 54 (0.1) | 2·0 |
| L03 | Cellulitis | 1,169 (3.0) | 513 (1.2) | 2·0 |
| H54 | Blindness and low vision | 614 (1.6) | 430 (1.0) | 1·9 |
| E53 | Deficiency of other B group vitamins | 529 (1.4) | 327 (0.8) | 1·9 |
| Z60 | Problems related to social environment | 229 (0.6) | 118 (0.3%) | 1·8 |
| G20 | Parkinson's disease | 562 (1.4) | 276 (0.7) | 1·8 |
| R55 | Syncope and collapse | 792 (2.0) | 437 (1.0) | 1·8 |
| S22 | Fracture of rib(s), sternum and thoracic spine | 310 (0.8) | 107 (0.3) | 1·8 |
| K59 | Other functional intestinal disorders | 3,344 (8.6) | 1,738 (4.1) | 1·8 |
| N17 | Acute renal failure | 11,278 (28.8) | 5,557 (13.2) | 1·8 |
| L89 | Decubitus ulcer | 803 (2.1) | 255 (0.6) | 1·7 |
| Z22 | Carrier of infectious disease | 86 (0.2) | 33 (0.1) | 1·7 |
| B95 | Streptococcus and staphylococcus as the cause of diseases classified to other chapters | 744 (1.9) | 907 (2.2) | 1·7 |
| L97 | Ulcer of lower limb, not elsewhere classified | 678 (1.7) | 316 (0.8) | 1.6 |
| R44 | Other symptoms and signs involving general sensations and perceptions | 101 (0.3) | 58 (0.1) | 1·6 |
| K26 | Duodenal ulcer | 264 (0.7) | 167 (0.4) | 1·6 |
| I95 | Hypotension | 5,002 (12.8) | 2,283 (5.4) | 1·6 |
| N19 | Unspecified renal failure | 44 (0.1) | 40 (0.1) | 1·6 |
| A41 | Other septicemia | 2,250 (5.8) | 1,397 (3.3) | 1·6 |
| Z87 | Personal history of other diseases and conditions | 10,882 (27.8) | 5,216 (12.4) | 1·5 |
| J96 | Respiratory failure, not elsewhere classified | 8,704 (22.3) | 4,216 (10.0) | 1·5 |
| M19 | Other arthrosis | 6,824 (17.5) | 5,533 (13.1) | 1·5 |
| G40 | Epilepsy | 626 (1.6) | 562 (1.3) | 1·5 |
| M81 | Osteoporosis without pathological fracture | 2,859 (7.3) | 2,146 (5.1) | 1·4 |
| S72 | Fracture of femur | 328 (0.8) | 128 (0.3) | 1·4 |
| S32 | Fracture of lumbar spine and pelvis | 213 (0.5) | 66 (0.2) | 1·4 |
| E16 | Other disorders of pancreatic internal secretion | 112 (0.3) | 84 (0.2) | 1·4 |
| R94 | Abnormal results of function studies | 587 (1.5) | 497 (1.2) | 1·4 |
| N18 | Chronic renal failure | 18,395 (47.0) | 9,854 (23.4) | 1·4 |
| R33 | Retention of urine | 1,944 (5.0) | 1,013 (2.4) | 1·3 |
| R69 | Unknown and unspecified causes of morbidity | 2 (0.0) | 4 (0.0) | 1·3 |
| N28 | Other disorders of kidney and ureter, not elsewhere classified | 1,405 (3.6) | 825 (2.0) | 1·3 |
| R32 | Unspecified urinary incontinence | 385 (1.0) | 155 (0.4) | 1·2 |
| G31 | Other degenerative diseases of nervous system, not elsewhere classified | 547 (1.4) | 282 (0.7) | 1·2 |
| Y95 | Nosocomial condition | 10 (0.0) | 3 (0.0) | 1·2 |
| S09 | Other and unspecified injuries of head | 103 (0.3) | 28 (0.1) | 1·2 |
| R45 | Symptoms and signs involving emotional state | 182 (0.5) | 86 (0.2) | 1·2 |
| G45 | Transient cerebral ischemic attacks and related syndromes | 264 (0.7) | 172 (0.4) | 1·2 |
| Z74 | Problems related to care-provider dependency | 465 (1.2) | 141 (0.3) | 1·1 |
| M79 | Other soft tissue disorders, not elsewhere classified | 1,129 (2.9) | 796 (1.9) | 1·1 |
| W06 | Fall involving bed | 4 (0.0) | 1 (0.0) | 1.1 |
| S01 | Open wound of head | 212 (0.5) | 81 (0.2) | 1.1 |
| A04 | Other bacterial intestinal infections | 298 (0.8) | 136 (0.3) | 1·1 |
| A09 | Diarrhea and gastroenteritis of presumed infectious origin | 36 (0.1) | 20 (0.0) | 1.1 |
| J18 | Pneumonia, organism unspecified | 4,149 (10.6) | 2,246 (5.3) | 1·1 |
| J69 | Pneumonitis due to solids and liquids | 633 (1.6) | 228 (0.5) | 1·0 |
| R47 | Speech disturbances, not elsewhere classified | 535 (1.4) | 408 (1.0) | 1·0 |
| E55 | Vitamin D deficiency | 1,234 (3.2) | 1,051 (2.5) | 1·0 |
| Z93 | Artificial opening status | 255 (0.7) | 90 (0.2) | 1·0 |
| R63 | Symptoms and signs concerning food and fluid intake | 760 (1.9) | 404 (1.0) | 0·9 |
| H91 | Other hearing loss | 2,342 (6.0) | 1,563 (3.7) | 0·9 |
| W10 | Fall on and from stairs and steps | 2 (0.0) | 4 (0.0) | 0·9 |
| W01 | Fall on same level from slipping, tripping and stumbling | 24 (0.1) | 6 (0.0) | 0·9 |
| E05 | Thyrotoxicosis [hyperthyroidism] | 450 (1.2) | 333 (0.8) | 0·9 |
| M41 | Scoliosis | 431 (1.1) | 363 (0.9) | 0·9 |
| R13 | Dysphagia | 1,588 (4.1) | 809 (1.9) | 0·8 |
| Z99 | Dependence on enabling machines and devices | 2,922 (7.5) | 673 (1.6) | 0·8 |
| M80 | Osteoporosis with pathological fracture | 103 (0.3) | 28 (0.1) | 0·8 |
| K92 | Other diseases of digestive system | 1,548 (4.0) | 597 (1.4) | 0·8 |
| I63 | Cerebral infarction | 613 (1.6) | 686 (1.6) | 0·8 |
| N20 | Calculus of kidney and ureter | 343 (0.9) | 290 (0.7) | 0·7 |
| F10 | Mental and behavioral disorders due to use of alcohol | 711 (1.8) | 1,122 (2.7) | 0·7 |
| Y84 | Other medical procedures as the cause of abnormal reaction of the patient | 22 (0.1) | 6 (0.0) | 0·7 |
| R00 | Abnormalities of heartbeat | 2,441 (6.2) | 2,056 (4.9) | 0·7 |
| J22 | Unspecified acute lower respiratory infection | 8 (0.0) | 6 (0.0) | 0·7 |
| Z73 | Problems related to life-management difficulty | 41 (0.1) | 9 (0.0) | 0·6 |
| R79 | Other abnormal findings of blood chemistry | 2,058 (5.3) | 1,069 (2.5) | 0·6 |
| Z91 | Personal history of risk-factors, not elsewhere classified | 3,513 (9.0) | 1,463 (3.5) | 0·5 |
| S51 | Open wound of forearm | 72 (0.2) | 17 (0.0) | 0·5 |
| F32 | Depressive episode | 4,967 (12.7) | 4,252 (10.1) | 0·5 |
| M48 | Spinal stenosis (secondary code only) | 1,129 (2.9) | 837 (2.0) | 0·5 |
| E83 | Disorders of mineral metabolism | 3,187 (8.2) | 1,984 (4.7) | 0·4 |
| M15 | Polyarthritis | 292 (0.7) | 205 (0.5) | 0·4 |
| D64 | Other anemias | 7,615 (19.5) | 3,467 (8.2) | 0·4 |
| L08 | Other local infections of skin and subcutaneous tissue | 35 (0.1) | 19 (0.0) | 0·4 |
| R11 | Nausea and vomiting | 605 (1.5) | 328 (0.8) | 0·3 |
| K52 | Other noninfective gastroenteritis and colitis | 519 (1.3) | 316 (0.8) | 0·3 |
| R50 | Fever of unknown origin | 242 (0.6) | 172 (0.4) | 0·1 |
| Data presented as n (%).  Abbreviation: M-TEER, mitral valve transcatheter edge-to-edge repair. | | | | |

## Table S2. Baseline Characteristics for Patients Undergoing M-TEER From 2017 Through 2021

|  | **2017** | **2018** | **2019** | **2020** | **2021** |
| --- | --- | --- | --- | --- | --- |
|  | **N=5,118** | **N=6,138** | **N=8,833** | **N=8,707** | **N=10,301** |
| Age, years | 80.6 ± 7.7 | 80.4 ± 7.6 | 80.1 ± 7.4 | 79.5 ± 7.4 | 79.6 ± 7.2 |
| Sex, male | 2,681 (52.4) | 3,336 (54.3) | 4,758 (53.9) | 4,688 (53.8) | 5,489 (53.3) |
| Race |  |  |  |  |  |
| White | 4,518 (88.3%) | 5,448 (88.8) | 7,680 (86.9) | 7,591 (87.2) | 8,916 (86.6) |
| Black | 303 (5.9) | 348 (5.7) | 604 (6.8) | 615 (7.1) | 768 (7.5) |
| Other | 297 (5.8) | 342 (5.6) | 549 (6.2) | 501 (5.8) | 617 (6.0) |
| Congestive heart failure | 4,489 (87.7) | 5,427 (88.4) | 7,959 (90.1) | 7,850 (90.2) | 9,183 (89.1) |
| Cardiac arrhythmias | 3,774 (73.7) | 4,584 (74.7) | 6,606 (74.8) | 6,464 (74.2) | 7,566 (73.4) |
| Atrial Fibrillation | 3,120 (61.0) | 3,865 (63.0) | 5,482 (62.1) | 5,343 (61.4) | 6,336 (61.5) |
| Pulmonary circulation disorders | 2,286 (44.7) | 2,641 (43.0) | 3,701 (41.9) | 3,462 (39.8) | 3,878 (37.6) |
| Peripheral vascular disorders | 1,355 (26.5) | 1,558 (25.4) | 2,296 (26.0) | 2,274 (26.1) | 2,431 (23.6) |
| Hypertension | 4,027 (78.7) | 5,029 (81.9) | 7,395 (83.7) | 7,291 (83.7) | 8,506 (82.6) |
| Paralysis | 59 (1.2) | 61 (1.0) | 74 (0.8) | 90 (1.0) | 88 (0.9) |
| Other neurological disorders | 403 (7.9) | 477 (7.8) | 715 (8.1) | 741 (8.5) | 867 (8.4) |
| Chronic pulmonary disease | 1,816 (35.5) | 2,139 (34.8) | 2,880 (32.6) | 2,830 (32.5) | 3,092 (30.0) |
| Diabetes, uncomplicated | 905 (17.7) | 894 (14.6) | 1,301 (14.7) | 1,214 (13.9) | 1,354 (13.1) |
| Diabetes, complicated | 1,112 (21.7) | 1,374 (22.4) | 2,050 (23.2) | 2,078 (23.9) | 2,315 (22.5) |
| Hypothyroidism | 1,260 (24.6) | 1,457 (23.7) | 2,242 (25.4) | 2,095 (24.1) | 2,439 (23.7) |
| Renal failure | 2,389 (46.7) | 2,944 (48.0) | 4,171 (47.2) | 4,145 (47.6) | 4,780 (46.4) |
| Liver disease | 228 (4.5) | 305 (5.0) | 473 (5.4) | 488 (5.6) | 618 (6.0) |
| Peptic ulcer disease excluding bleeding | 94 (1.8) | 79 (1.3) | 140 (1.6) | 117 (1.3) | 122 (1.2) |
| AIDS/HIV | 6 (0.1) | 7 (0.1) | 6 (0.1) | 9 (0.1) | 8 (0.1) |
| Lymphoma | 73 (1.4) | 69 (1.1) | 150 (1.7) | 113 (1.3) | 163 (1.6) |
| Metastatic cancer | 40 (0.8) | 54 (0.9) | 107 (1.2) | 91 (1.0) | 99 (1.0) |
| Solid tumor without metastasis | 192 (3.8) | 230 (3.7) | 416 (4.7) | 368 (4.2) | 433 (4.2) |
| Rheumatoid arthritis/collagen vascular disorder | 265 (5.2) | 329 (5.4) | 503 (5.7) | 447 (5.1) | 511 (5.0) |
| Coagulopathy | 742 (14.5) | 883 (14.4) | 1,243 (14.1) | 1,243 (14.3) | 1,394 (13.5) |
| Obesity | 717 (14.0) | 956 (15.6) | 1,489 (16.9) | 1,566 (18.0) | 1,893 (18.4) |
| Weight loss | 498 (9.7) | 592 (9.6) | 863 (9.8) | 815 (9.4) | 873 (8.5) |
| Fluid and electrolyte disorders | 1,717 (33.5) | 2,118 (34.5) | 3,031 (34.3) | 3,108 (35.7) | 3,383 (32.8) |
| Blood Loss anemia | 141 (2.8) | 163 (2.7) | 249 (2.8) | 191 (2.2) | 199 (1.9) |
| Deficiency anemia | 533 (10.4) | 661 (10.8) | 995 (11.3) | 989 (11.4) | 1,129 (11.0) |
| Alcohol abuse | 85 (1.7) | 112 (1.8) | 174 (2.0) | 197 (2.3) | 238 (2.3) |
| Drug abuse | 40 (0.8) | 79 (1.3) | 107 (1.2) | 114 (1.3) | 119 (1.2) |
| Psychoses | 21 (0.4) | 12 (0.2) | 21 (0.2) | 30 (0.3) | 35 (0.3) |
| Depression | 725 (14.2) | 820 (13.4) | 1,240 (14.0) | 1,216 (14.0) | 1,401 (13.6) |
| Hospital Frailty Risk Score | 6.6 ± 6.9 | 6.7 ± 6.8 | 6.5 ± 6.8 | 6.4 ± 6.7 | 5.9 ± 6.4 |
| Frailty risk |  |  |  |  |  |
| Low | 2,683 (52.4) | 3,182 (51.8) | 4,691 (53.1) | 4,652 (53.4) | 5,859 (56.9) |
| Intermediate | 1,849 (36.1) | 2,252 (36.7) | 3,086 (34.9) | 3,065 (35.2) | 3,435 (33.3) |
| High | 586 (11.4) | 704 (11.5) | 1,056 (12.0) | 990 (11.4) | 1,007 (9.8) |
| Frail | 2,435 (47.6) | 2,956 (48.2) | 4,142 (46.9) | 4,055 (46.6) | 4,442 (43.1) |
| Data presented as mean ± standard deviation or n (%), where applicable.  Abbreviations: AIDS, acquired immunodeficiency syndrome; HIV, Human immunodeficiency virus; M-TEER, mitral valve transcatheter edge-to-edge repair. | | | | | |

## Table S3. Baseline Characteristics for Patients Undergoing Surgical Mitral Valve Repair From 2017 Through 2021

|  | **2017** | **2018** | **2019** | **2020** | **2021** |
| --- | --- | --- | --- | --- | --- |
|  | **N=9,910** | **N=8,600** | **N=8,671** | **N=7,228** | **N=7,709** |
| Age, years | 73.7 ± 5.7 | 73.5 ± 5.6 | 73.3 ± 5.4 | 73.0 ± 5.3 | 73.0 ± 5.3 |
| Sex, male | 5,858 (59.1) | 5,134 (59.7) | 5,296 (61.1) | 4,428 (61.3) | 4,602 (59.7) |
| Race |  |  |  |  |  |
| White | 8,770 (88.5) | 7,535 (87.6) | 7,553 (87.1) | 6,309 (87.3) | 6,687 (86.7) |
| Black | 513 (5.2) | 451 (5.2) | 454 (5.2) | 374 (5.2) | 363 (4.7) |
| Other | 627 (6.3) | 614 (7.1) | 664 (7.7) | 545 (7.5) | 659 (8.5) |
| Congestive heart failure | 5,945 (60.0) | 5,157 (60.0) | 5,065 (58.4) | 4,264 (59.0) | 4,436 (57.5) |
| Cardiac arrhythmias | 5,682 (57.3) | 4,979 (57.9) | 4,858 (56.0) | 4,139 (57.3) | 4,373 (56.7) |
| Atrial Fibrillation | 4,744 (47.9) | 4,134 (48.1) | 4,053 (46.7) | 3,438 (47.6) | 3,556 (46.1) |
| Pulmonary circulation disorders | 2,887 (29.1) | 2,476 (28.8) | 2,318 (26.7) | 1,960 (27.1) | 2,007 (26.0) |
| Peripheral vascular disorders | 1,443 (14.6) | 1,252 (14.6) | 1,178 (13.6) | 1,065 (14.7) | 1,105 (14.3) |
| Hypertension | 5,180 (52.3) | 4,701 (54.7) | 4,635 (53.5) | 3,938 (54.5) | 4,076 (52.9) |
| Paralysis | 93 (0.9) | 67 (0.8) | 72 (0.8) | 57 (0.8) | 63 (0.8) |
| Other neurological disorders | 490 (4.9) | 396 (4.6) | 385 (4.4) | 360 (5.0) | 366 (4.7) |
| Chronic pulmonary disease | 2,249 (22.7) | 1,863 (21.7) | 1,875 (21.6) | 1,486 (20.6) | 1,538 (20.0) |
| Diabetes, uncomplicated | 1,187 (12.0) | 907 (10.5) | 810 (9.3) | 629 (8.7) | 590 (7.7) |
| Diabetes, complicated | 1,499 (15.1) | 1,314 (15.3) | 1,303 (15.0) | 1,027 (14.2) | 1,043 (13.5) |
| Hypothyroidism | 1,664 (16.8) | 1,383 (16.1) | 1,390 (16.0) | 1,133 (15.7) | 1,208 (15.7) |
| Renal failure | 2,422 (24.4) | 2,014 (23.4) | 2,003 (23.1) | 1,691 (23.4) | 1,768 (22.9) |
| Liver disease | 295 (3.0) | 223 (2.6) | 276 (3.2) | 277 (3.8) | 281 (3.6) |
| Peptic ulcer disease excluding bleeding | 105 (1.1) | 71 (0.8) | 63 (0.7) | 55 (0.8) | 56 (0.7) |
| AIDS/HIV | 8 (0.1) | 10 (0.1) | 7 (0.1) | 4 (0.1) | 5 (0.1) |
| Lymphoma | 69 (0.7) | 48 (0.6) | 62 (0.7) | 40 (0.6) | 42 (0.5) |
| Metastatic cancer | 46 (0.5) | 36 (0.4) | 31 (0.4) | 24 (0.3) | 32 (0.4) |
| Solid tumor without metastasis | 210 (2.1) | 181 (2.1) | 200 (2.3) | 160 (2.2) | 160 (2.1) |
| Rheumatoid arthritis/collagen vascular disorder | 355 (3.6) | 340 (4.0) | 291 (3.4) | 240 (3.3) | 255 (3.3) |
| Coagulopathy | 1,348 (13.6) | 1,128 (13.1) | 1,163 (13.4) | 1,030 (14.3) | 1,170 (15.2) |
| Obesity | 1,793 (18.1) | 1,558 (18.1) | 1,557 (18.0) | 1,370 (19.0) | 1,462 (19.0) |
| Weight loss | 553 (5.6) | 457 (5.3) | 475 (5.5) | 425 (5.9) | 427 (5.5) |
| Fluid and electrolyte disorders | 2,020 (20.4) | 1,725 (20.1) | 1,717 (19.8) | 1,592 (22.0) | 1,591 (20.6) |
| Blood loss anemia | 122 (1.2) | 111 (1.3) | 94 (1.1) | 74 (1.0) | 70 (0.9) |
| Deficiency anemia | 430 (4.3) | 397 (4.6) | 354 (4.1) | 327 (4.5) | 350 (4.5) |
| Alcohol abuse | 268 (2.7) | 247 (2.9) | 231 (2.7) | 211 (2.9) | 222 (2.9) |
| Drug abuse | 83 (0.8) | 100 (1.2) | 108 (1.2) | 114 (1.6) | 73 (0.9) |
| Psychoses | 26 (0.3) | 21 (0.2) | 21 (0.2) | 20 (0.3) | 21 (0.3) |
| Depression | 1,056 (10.7) | 899 (10.5) | 940 (10.8) | 817 (11.3) | 939 (12.2) |
| Hospital Frailty Risk Score | 3.3 ± 4.6 | 3.3 ± 4.6 | 3.2 ± 4.4 | 3.3 ± 4.5 | 3.1 ± 4.2 |
| Frailty risk |  |  |  |  |  |
| Low | 7,663 (77.3) | 6,701 (77.9) | 6,835 (78.8) | 5,550 (76.8) | 6,102 (79.2) |
| Intermediate | 1,935 (19.5) | 1,609 (18.7) | 1,605 (18.5) | 1,480 (20.5) | 1,417 (18.4) |
| High | 312 (3.1) | 290 (3.4) | 231 (2.7) | 198 (2.7) | 190 (2.5) |
| Frail | 2,247 (22.7) | 1,899 (22.1) | 1,837 (21.2) | 1,678 (23.2) | 1,607 (20.9) |
| Data presented as mean standard deviation or n (%), where applicable.  Abbreviations: AIDS, acquired immunodeficiency syndrome; HIV, Human immunodeficiency virus. | | | | | |

## Figure S1. Annual Change in Comorbidities (Odds Ratio per Year)

Each point represents the annual odds ratio (OR per year) for the presence of each comorbidity from 2017–2021, estimated using logistic regression with year as a continuous predictor. Horizontal lines indicate 95% confidence intervals.

Abbreviations: AIDS, acquired immunodeficiency syndrome; CHF, congestive heart failure; Circ, circulation; COPD, chronic obstructive pulmonary disorder; F&E, fluid and electrolyte; HIV, human immunodeficiency virus; M-TEER, mitral valve transcatheter edge-to-edge repair; PVD, pulmonary vascular disorder.

## Figure S2. Absolute (Risk Difference) Changes in Comorbidity Prevalence

The risk difference per year represents the corresponding absolute yearly change in prevalence, calculated from the marginal effect of calendar year in the logistic regression analysis.

Abbreviations: AIDS, acquired immunodeficiency syndrome; CHF, congestive heart failure; Circ, circulation; COPD, chronic obstructive pulmonary disorder; F&E, fluid and electrolyte; HIV, human immunodeficiency virus; M-TEER, mitral valve transcatheter edge-to-edge repair; PVD, pulmonary vascular disorder.
